# Biomarkers of Nutrition and Health: New Tools for New Approaches

**DOI:** 10.3390/nu11051092

**Published:** 2019-05-16

**Authors:** Catalina Picó, Francisca Serra, Ana María Rodríguez, Jaap Keijer, Andreu Palou

**Affiliations:** 1 Laboratory of Molecular Biology, Nutrition and Biotechnology (Group of Nutrigenomics and Obesity), CIBER de Fisiopatología de la Obesidad y Nutrición (CIBERobn) and Instituto de Investigación Sanitaria Illes Balears (IdISBa), University of the Balearic Islands, ES-07122 Palma de Mallorca, Spain; cati.pico@uib.es (C.P.); amrodriguez@uib.es (A.M.R.); andreu.palou@uib.es (A.P.); 2 Human and Animal Physiology, Wageningen University, PO Box 338, 6700 AH Wageningen, The Netherlands; jaap.keijer@wur.nl

**Keywords:** food intake assessment, integrative biomarkers, omics technologies, precision nutrition

## Abstract

A main challenge in nutritional studies is the valid and reliable assessment of food intake, as well as its effects on the body. Generally, food intake measurement is based on self-reported dietary intake questionnaires, which have inherent limitations. They can be overcome by the use of biomarkers, capable of objectively assessing food consumption without the bias of self-reported dietary assessment. Another major goal is to determine the biological effects of foods and their impact on health. Systems analysis of dynamic responses may help to identify biomarkers indicative of intake and effects on the body at the same time, possibly in relation to individuals’ health/disease states. Such biomarkers could be used to quantify intake and validate intake questionnaires, analyse physiological or pathological responses to certain food components or diets, identify persons with specific dietary deficiency, provide information on inter-individual variations or help to formulate personalized dietary recommendations to achieve optimal health for particular phenotypes, currently referred as “precision nutrition.” In this regard, holistic approaches using global analysis methods (omics approaches), capable of gathering high amounts of data, appear to be very useful to identify new biomarkers and to enhance our understanding of the role of food in health and disease.

## 1. Introduction

The nutritional status of an individual reflects the extent to which their physiological needs of nutrients have been covered at a particular life stage. When the nutrients to support daily body needs and metabolic demands are consumed in a balanced manner, without insufficiency or excess, the person presents an optimal nutritional status that favours growth, development, appropriate cell/tissue turnovers and global health.

Dietary assessment and nutritional status are traditionally measured by means of dietary intake data, such as 24-h dietary recalls, food records or food frequency questionnaires [1]. Even though recent technological advances, including image analysis software, to collect dietary information or to process dietary data, have improved dietary assessment, food-intake based methods have some inherent limitations, such as:

Subjective nature of data collection tools. People do not always remember everything they have consumed or are not able to recall all foods eaten or their specific ingredients/components or they may have difficulty estimating portion sizes accurately [2]. This combination of factors determines measurement errors in dietary assessment. Moreover, individuals often underreport dietary intake, particularly when reporting intakes which have higher social desirability and, for those who have a history of dieting and being overweight, reflect greater eating restraint [3,4].

Limitations of food composition tables. Some nutrients, such as the vast majority of trace elements, are not sufficiently characterised in food composition tables and, therefore, nutritional status cannot be assessed correctly based on intake [5]. This is also the case for certain fat-soluble vitamins. Fats and oils constitute the main nutritional source of vitamin E; however, the content of this vitamin varies depending on the type of oil, its processing, the addition of antioxidants and its shelf life, all of which cannot be characterised in a dietary assessment. On the other hand, the nutritional content of food is neither consistent nor uniform and food composition databases may not reflect the characteristics of the products currently commercialised. They generally lag behind current eating patterns, for example, the tendency for whole grain products is poorly reflected.

Factors influencing nutrient absorption. Certain nutrients have feedback control mechanisms that increase or decrease the efficiency of absorption depending on nutritional status; for example, an individual with a low nutritional calcium status will absorb calcium more efficiently [6]. Certain food combinations can affect absorption; for example, the fibre content of a meal may decrease the availability of food carotenoids [7], whereas the vitamin C content promote iron absorption when ingested at the same time [8]. Vitamin D was shown to be better available from milk than from solid food [9]. The extent of cooking of foods may also influence composition, including nutrient content and absorption, such is the case of vitamin B6 and vitamin C [6]. Finally, the degree of processing may affect absorption, since micronutrients can be associated with proteins that facilitate their bioavailability, which has been shown, for example, for calcium [10] and zinc [11] or which are better available in their native form (e.g., heme-iron, Fe^3+^) [12]. These factors are generally not considered, because dietary questionnaires do not include enough detail on how food is prepared or processed and do not capture information about foods eaten together.

## 2. Biomarkers of Nutritional Status

The limitation of dietary assessment to estimate nutritional status determines the need for analytical determinants that can objectively and accurately quantify nutritional status. Biomarkers provide a more proximal measure of nutrient status than dietary intake. Generally speaking, a nutritional biomarker is a characteristic that can be objectively measured in different biological samples and can be used as an indicator of nutritional status with respect to the intake or metabolism of dietary constituents [6]. Examples of suggested nutritional biomarkers are shown in Table 1.

The biochemical analysis of a reference metabolite that indicates the bioavailability of a nutrient is an objective result to assess nutritional status, which entails lower methodological error and detects deficiency states more precisely than dietary assessment. Such biomarkers are generally based on pronounced changes observed in one parameter. They are clinically useful, in particular to detect deficiencies in support of medical treatment. Analysis of folate, iron and vitamin B12 but also copper and zinc, is useful to identify potential nutritional causes of anaemia [13]. The development of biomarkers faithfully representing the nutritional status for those micronutrients is clearly justified by their usefulness in medicine.

Clinical biomarkers are focused on diagnosis of a disease state. In most cases these are independent of nutrition but comprise the best parameter that reflects a certain disease. In case of metabolic diseases, overlap may occur, for example, this is the case for circulating lipid profiles, urea levels in blood or urine and so forth. In most instances, clinical diagnosis of disease differs from nutritional biology, that focuses on health, that is, whether the nutritional status is such that it supports health or not. The latter can also entail mild subclinical deficiencies as well as moderate excess. Especially in those cases, the combination of both methodologies, dietary assessment by food questionnaires with biochemical measures, can provide a useful tool for estimating the exposure to a particular nutrient of interest and assessing health risks. This combination may eliminate some of the errors associated with each type methods to assess nutritional status [6].

**Table 1:** Examples of suggested nutritional biomarkers related with exposure and/or effects of macronutrients, food or dietary patterns, in samples obtained with non-invasive or minimally invasive techniques. Some representative references are provided for each candidate biomarker.

Proposed Biomarker	Sample Type	Intended Use (As Nutritional Biomarker)	References
Alkylresorcinols	Plasma	Whole-grain food consumption	Original research [14,15] Reviewed in Reference [16]
Allyl methyl sulfoxide (AMSO) or allyl methyl sulfone (AMSO_2_)	Urine	Intake of garlic	Original research [17] BFIRev ** [18]
Allyl methyl sulphide (AMS)	Urine/breath	Intake of garlic	Original research [17,19,20] BFIRev [18]
Arbutin	Plasma	Pear intake	Original research [21] BFIRev [22]
Carotenoids	Plasma	Fruit and vegetable intake	Systematic review and meta-analysis [23]
Carotenoids with Vitamin C	Plasma/serum	Fruit and vegetable intake Combined marker (suggested as better biomarker than carotenoids or vitamin C alone)	Reviewed in Reference [24]
Creatine	Serum	Intake of meat and fish	Reviewed in Reference [25]
Creatinine	Urine	Intake of meat and fish	Reviewed in Reference [25]
Daidzein	Urine/plasma	Intake of soy or soy-based products	Systematic review [26]
Dyhydrocaffeic acid derivatives	Urine	Acute and habitual exposure to coffee	Original research [27,28,29] Reviewed in Reference [30]
Erythronic acid, alone or with fructose and/or sucrose	Urine	Sugar intake Combined marker	Original research [31]
Genistein	Urine/plasma	Intake of soy or soy-based products	Systematic review [26]
Homocysteine	Plasma	One carbon metabolism and folate status	Reviewed in References [32,33]
Hydroxylated and sulfonated metabolites of esculeogenin B	Urine	Intake of tomato juice	Original research [34]
1-Methylhistidine	Urine	Meat and oily fish consumption	Original research [27,35,36] Reviewed in References [30,37]
n-3 fatty acids: docosahexaenoic acid (DHA)	Blood: erythrocytes or platelets	DHA status	Systematic review [38]
n-3 fatty acids: DHA (as phospholipid)	Plasma	DHA status	Systematic review [38]
n-3 fatty acids: eicosapentaenoic acid (EPA as phospholipid)	Plasma	EPA status	Systematic review [38]
*N*-acetyl-*S*-(2carboxypropyl)cysteine (CPMA)	Urine	Intake of onion and garlic	Original research [39] BFIRev [18]
Nitrogen*	Urine (24h)	Protein intake	Reviewed in Reference [40]
*O*-acetylcarnitine	Urine	Red-meat consumption	Original research [41] Reviewed in Reference [42]
Pentadecanoic acid (C15:0)	Plasma/serum	Total dairy fat intake	Reviewed in Reference [43]
Phenylacetylglutamine	Urine	Vegetable intake	Original research [41] Reviewed in Reference [30]
Phloretin	Urine	Apple intake	Original research [44,45] BFIRev [22]
Phloretin glucuronide	Urine	Apple intake	Original research [46,47] BFIRev [22]
Proline betaine	Urine	Acute and habitual citrus exposure	Original research [27,48,49] Reviewed in Reference [30]
*S*-allylcysteine (SAC)	Plasma	Intake of garlic	Original research [19] BFIRev [18]
*S*-allylmercapturic acid (ALMA)	Urine	Intake of garlic	Original research [50] BFIRev [18]
Urolithin B	Urine	Intake of ellagitannins (present in fruits as strawberries, raspberries and walnuts and oak-aged red wine, among others)	Original research [51]

* Nitrogen in 24 h urine is an already substantially validated biomarker of protein intake. ** BFIRev: Biomarker of Food Intake Review. This type of review follows specific recent guidelines for the review, identification and/or validation of candidate biomarkers of food intake [52].

To understand the complex relationships between nutrition and health, different types of biomarkers are being used in nutritional studies: markers of exposure, of effect (or function) and of health/disease state [53].

Biomarkers of exposure. These include the different types of biomarkers used to evaluate dietary intake of nutrients, non-nutritive food components or dietary patterns. An example is the nitrogen in urine [40], which serves as a biomarker for protein intake. These types of biomarkers are of great interest, as their use can help to improve the categorization of subjects according to the exposure to a particular nutrient. They also serve as an objective indicator of compliance with a particular dietary regimen in intervention studies investigating the health effects of dietary modifications [54]. These biomarkers may not only reflect one nutrient but may also be associated with a dietary pattern or food group, for example, the plasma concentration of alkylresorcinol is considered a biomarker of the intake of whole grains [14] and the combination of sucrose or fructose with erythronic acid is a urinary biomarker for sugar intake [31]. Urine/plasma genistein and daidzein are also biomarkers for soy or soy-based product intake [26], while robust information for markers of other legumes is still lacking. In this sense a combination of markers may better reflect a food category, for example vitamin C and carotenoids together may be more accurate that either of these fruit and vegetable biomarkers alone [24].

Biomarkers of effects. These are biomarkers that are related to a target function or biological response. Thus, not only do they reflect intake but also nutrient metabolism and, possibly, effects on physiological or disease processes. It is important to note that a biomarker may not reflect the effect of a single nutrient but the interactions of various nutrients. For example, some of the biomarkers of the metabolism of one carbon compounds such as homocysteine, which reflect not only nutritional intake but also various metabolic processes related with pathological or physiological conditions [32,33].

Biomarkers of health/disease and physiological status. These are biomarkers which indicate an end-point, relate to a state of health and/or disease risk. These markers reflect the different intermediate disease phenotypes or the severity of the disease and are widely used in clinical practice. For example, plasma levels of fasting glucose are associated with insulin sensitivity and diabetes or plasma cholesterol and triglycerides are linked to cardiovascular disease. Nutritional biomarker research is not focused on identification and characterization of diseases or treatment prognostics, which are areas of intense development.

## 3. Current Challenges in the Development of Health Biomarkers

The development of health/disease biomarkers was driven by medical needs and has largely been directed towards identifying and quantifying disease states or progression, rather than assessing and quantifying the health status of an individual. However, the main objective of diet and nutrition is to promote and maintain optimal health. Therefore, it is highly relevant to have biomarkers of very early stages of alterations that may ultimately progress to disease, even before what may be considered the onset of the disease. Such biomarkers can be considered health and/or prevention markers rather than disease markers. Pre-disease physiological alterations are likely to be associated with pre-disease alterations in homeostatic balance and may be identified when the homeostatic response to a particular environmental or nutritional aggression is examined [55]. These biomarkers represent a new approach to biomarkers that reflect maintenance of physiological integrity and function. In this context, health-promoting food components support or even optimise, a healthy physiology, preventing or delaying initiation of a disease state or a loss of physiological function, including cognitive function. Because of the multifaceted nature of homeostasis, nutrigenomic technologies, which analyse functional genomic responses on a genome-wide scale applied to the field of nutrition, have been particularly valuable and will continue to be so, for the identification, characterisation and validation of health biomarkers.

Human health is based on a complex network of interactions between pathways, processes and molecules, implying interactive mechanisms and across different cells, tissues and organs. Various biochemical and physiological mechanisms are responsible for maintaining health in an environment that is constantly changing, as a result of, for example, diet, infections, temperature, exercise and various other stressors. In good health, the mechanisms that maintain homeostasis are able to effectively buffer the different challenges that individuals are subjected to. The adaptation response defines the so-called phenotypic flexibility [56]. The way to the disease starts when and where these adaptive processes and regulatory networks fail. Lifestyle and other conditions, both environmental and internal, can reduce the robustness and elasticity of these mechanisms. Then the homeostatic machinery becomes less effective and produces negative side effects, which can occur from the molecular to the whole-body level. An example is excessive accumulation of lipids in the liver, which can be a consequence of the diminished capacity of adipose tissue for lipid storage [57]. This results in an organism that has lost its ability to react adequately to external challenges, which further aggravates the situation, for example by the development of insulin resistance. This loss of an adequate physiological performance, resulting in the inability to maintain healthy responses, can be the basis for the development of new biomarkers, reflecting dynamic responses, to assess health status and the capacity for its metabolic flexibility.

Biomarkers of health can guide policies related to food, nutrition and health. In fact, they would represent the basis for the substantiation of health claims on food. At present, the lack of robust nutritional biomarkers for many biological functions is a bottleneck that slows down innovation in the food industry. This is recognized and taken up by the scientific community. One example is BIOCLAIMS (FP7-244995), a collaborative research project carried out at the European level, which has established the principles to identify, establish and validate robust biomarkers to quantify the health status. Examining the influence of bioactive components of the diet on these biomarkers, provides the basis for evidence-based development of foods with reliable health properties that can contribute to a healthier diet and health in the long term. Other initiatives, such as PREVENTOMICS (DT-SFS-14-2018-818318), a project funded by the European Union’s program Horizon 2020 under the call ICT-04-2017—Personalized Nutrition, aims to use health biomarkers in dietary advice applications for consumers.

### The New Concept of Integrative Nutritional Biomarkers

**Figure 1:**
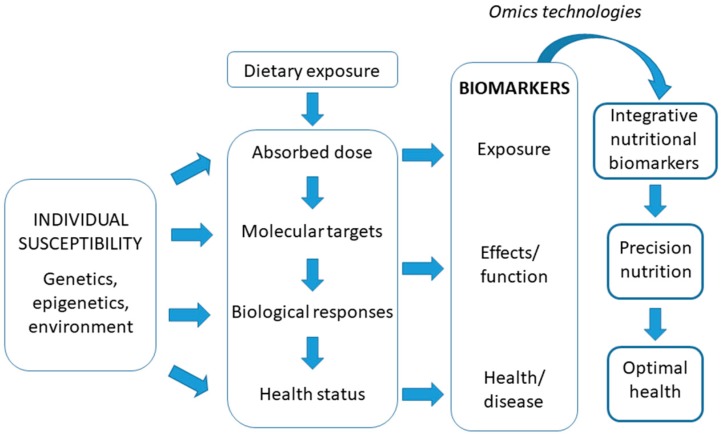
Integrative nutritional biomarkers and their interest in precision nutrition. Biomarkers of exposure include biological markers intended for the assessment of dietary food intake, whereas biomarkers of effect/function are related to target function or biological response. These biomarkers reflect not only the intake but also the metabolism of nutrients and, possibly, effects on disease processes. Biomarkers of health/disease are biomarkers of ultimate goal and indicative of improved health status and/or reduced risk of disease. Several factors (genetic, epigenetic, environment, etc.) can affect the individual response to dietary intake and its relation to health status. There is a great interest in the development of new types of nutritional biomarkers with an integrative trait, indicative of the intake and effects on the organism, including its relationship with the state of health/disease and omics technologies may play a relevant role.

In terms of nutrition, health biomarkers are the cornerstone of research that establishes the functional effects of nutrition on the health-disease relationship. Currently, given the complex relationship between food intake and health/disease status, a more integrative understanding of the concept of biomarker in relation to nutritional status and health is being developed, by focusing in nutritionally-regulated biomarkers of health. The concept is that intake is quantified, not only in terms of what is eaten but also in terms of the evoked biological response. As an example, circulating lipid profiles reflect intake but also depend on nutritional context, genotype and health status [58]. Similarly, a specific protein modification may be a physiological response that may also reflect intake. The development of such a new type of biomarker with an integrative trait, integrative nutritional biomarkers, recognizes the intimate connection between nutrition and metabolism. They could be indicative of both intake and of effects on the body and could even reflect health/disease state. Integrative nutritional biomarkers use the fact that nutrition and metabolism are intimately connected, which is considered an advantage rather than a hindrance and source of variation. They may be defined by a single parameter but more likely a set of directly connected parameters, for example a protein and its physiology and nutrient induced modifications or a spectrum of plasma lipids but can also consist of an integrating algorithm based on several parameters, each reflecting a particular aspect of metabolism and nutrient exposure and availability. Such biomarkers could be analytical indicators, which would be quantitative and acting as an intake (short- or long-term exposure) indicator and/or pointing to the status of a particular nutrient or food component and integrate the impact of intake on the body (effect). Integrative nutritional biomarkers could be used to quantify effective intakes and validate or complement intake questionnaires, shed light on physiological or pathological responses to certain food behaviours, monitor responses to therapeutic interventions that could be optimised and more personalised and provide information on inter-individual variations in response to the diet. Furthermore, they may help to formulate personalized dietary recommendations to achieve optimal health and wellness for particular phenotypes and genotypes, currently referred to as “precision nutrition” [59]. Inter-individual differences may have a genetic basis, for example associated with the presence of concrete polymorphisms, and/or also epigenetic basis, related with a particular genotype interaction with environmental characteristics (including diet) and life stage. Thus, interpretation of the meaning of biomarkers may require a holistic view (Figure 1). Biomarkers may reflect the effects of nutrient intake or a lack thereof and in certain cases, they can also act as an intermediate biomarker that indicates the potential risk of developing a pathology associated with either excess or deficit of the nutrient to which it is linked.

To date, there is still not a clear consensus regarding the requirements for nutritional biomarkers and the foundations needed to define optimal biomarkers for particular nutrients and their application is a subject of extensive research [60]. In this context, it has been proposed that biomarkers should meet the following criteria [61]: (a) they should be determined by solid, sensitive, reproducible methods, which should be highly specific and economically feasible; (b) their concentration in the biological sample must be sensitive enough to reflect possible changes, both in relation to the considered health status and to the dietary intervention; (c) they must be specific to the purpose for which they are used. It is also important that biomarkers are present in biological samples that are easily accessible and obtained using minimally invasive techniques. Other factors, such as age, gender, ethnicity, may be of interest depending on the purpose of use of the biomarkers.

The correct interpretation of a biomarker requires clearly defined standards of reference. Reference values are the values of an analyte in a reference population that is usually formed by a group of healthy individuals. If values show a normal distribution, the reference range is the population mean ±2 times the standard deviation, which is therefore the central interval of 95% of the distribution [62]. The interpretation of the results obtained in laboratory tests is based on the comparison made with said reference values. A value which deviates from said reference range does not necessarily imply that it is an abnormal value but it does mean that it has a greater probability to be associated with a deficit or excess and hence relatively closer to pathological values. Reference ranges may depend on the characteristics of the population, age and sex; they can also vary for arterial and venous blood, specific diets and so forth. Laboratory tests commonly used to assess nutritional status are well characterised, although to date, there are still some nutrients for which the normal range in healthy individuals has not been clearly defined. For example, serum 25-hydroxyvitamin D is widely recognised as a good marker for vitamin D status, reflecting its intake and endogenous synthesis [63]. The threshold of deficiency has been established between 25–50 nmol/L, associated with its effects on calcium and phosphate metabolism and bone health. However, the emergence of new physiological roles of vitamin D related to cardiovascular health seems to point to a certain benefit of higher concentrations in the general population [64]. Moreover, serum concentrations below the reference range are not necessarily associated with deficiency. The African-American population has, on average, lower circulating levels compared to the Caucasian population, even though the prevalence of osteoporosis and the occurrence of fractures are lower [65]. Thus, validated, sensitive and specific margins are required to assess the status of various nutrients and their effects and allow for correct classification of the nutritional status.

## 4. Sources of Biomarkers in Nutritional Studies

The most commonly used biological samples in nutritional epidemiology are blood-borne (plasma, serum, blood cells), excretion products (urine, faeces) or easily obtainable specimens (nails, saliva, hair), although in certain cases it may be relevant to have biopsies or solid tissue samples (muscle, adipose, skin).

The type of sample must be considered when processing blood samples: blood (collected with an anticoagulant and without removing any constituent), serum (allowing blood clotting and collecting the supernatant after subsequent centrifugation, which removes the clot and blood cells) or plasma (the aqueous fraction containing blood proteins, electrolytes and metabolites). The assessment of biomarkers in blood cells may also be appropriate, by analysing the respective fractions (erythrocytes and leukocytes, mainly). For example, the determination of the omega-3 index (eicosapentaenoic acid (EPA) and docosahexaenoic acid (DHA) content related to the total percentage of fatty acids) in erythrocyte membranes is considered a good biomarker of omega-3 fatty acid intake [38].

Peripheral blood cells (PBCs) are of particular interest because they are a source of transcriptome-based biomarkers and can be easily obtained using minimally invasive techniques. Some of the gene expression studies in blood cells are carried out in a specific subpopulation, peripheral blood mononuclear cells (PBMCs), including lymphocytes and monocytes, which are a reliable and homogeneous sample for transcriptome analysis [66]. Indeed, the PBMC transcriptome reflects the beneficial effects of a hyaluronic acid containing extract on articular health in humans [67]. Using preclinical models, we have also shown that PBMCs can faithfully reflect effects of dietary and environmental interventions in organs that are not accessible for analysis in healthy human subjects, including the liver [68,69,70] and hypothalamus [71]. It should be noted however, that the procedure for the isolation of PBMCs requires that a strict protocol is followed, which must be carried out immediately after blood collection to avoid ex vivo changes in gene expression profile. This can cause a number of logistical and technical problems, particularly when multicentre studies are involved. Existing alternative techniques include the PAXgene blood RNA system, which allows the extraction and stabilisation of the RNA of blood cells without additional handling [72]. This procedure offers a range of technical advantages, such as ease in collecting, storing and transporting samples, as well as reducing sample handling time, factors which facilitate standardisation and reproducibility. This makes it an attractive approach for use as a source of biomarkers in human nutritional studies [73]. The limitation in using total blood cells is that it does not allow the classification of specific cell populations. In addition, some studies have shown increased background noise and a reduction in responsiveness to stimuli (for example, in functional analysis) compared to the use of PBMCs [74]. However, it has been shown that there is a significant overlap in the gene expression profile between whole blood (using PAXgene tubes) and PBMCs [75] and therefore it could be expected that the identified biomarkers using PBMCs can be extended to total blood cells, which is more attractive for large scale human studies.

For certain applications, the blood from a finger or heel prick deposited and absorbed on paper, the so-called ‘dried blood spot’ technique, can be used for screening, for example, genetic screening of infants for phenylketonuria or to analyse certain hormonal or metabolites, such as fatty acid analysis [76]. Due to rapid technological developments, dried blood spot approaches are currently being developed to assess nutrient exposure [77], to identify nutrient-exposure associated risk markers [78] and to quantify markers for nutrition-related metabolic status [79], as well as disease risk markers [80].

A recent source for blood derived biomarkers are ‘extracellular vesicles’ (EVs), which is a collective term for cell-released, membranous structures. Recently, the International Society for Extracellular Vesicles (ISEV) updated guidelines of Minimal Information for Studies of Extracellular Vesicles (MISEV) to document specific EV-associated parameters, which is essential for their use as a source for biomarkers [81]. Circulating EVs can reflect specific tissues and provide an opportunity for biomarkers associated with tissues that are hardly accessible, such as the central nervous system [82,83]. For this reason, EV sampling has been endowed with the term ‘liquid biopsy’ [84]. They offer the potential for diagnosis and monitoring and, because they provide almost continuous circulating information based on blood sampling, can potentially be used in epidemiological investigations, for example directed at cardiovascular disease risk [85]. In fact, EV can be purified from a number of human body fluids including plasma, saliva and breast milk, which is particularly enriched in microRNAs (see Section 6.3.1).

Breast milk may be a source of biomarkers of the maternal nutritional and metabolic state [86,87]. A possible complicating factor is that the breast milk composition is not uniform and may be influenced by maternal, infant and environmental factors. Hence, a sampling protocol has been proposed to obtain an average sample [88].

Human breast milk is of particular relevance for analysis of the complex relationship between the maternal nutritional status and infant health [89,90]. Breast milk composition may affect infant growth and development and may have a strong impact on future metabolic health [91]. For example, animal studies have shown that lactation by obese, diabetic or malnourished mothers predisposes for metabolic disorders in the offspring [92]. Metabolome analysis has revealed changes in milk composition in rat dams exposed to moderate calorie restriction during lactation, which may be associated with the lower predisposition to obesity and the healthier phenotype described in the adult offspring [93]. However, the possible contribution and potential benefits of specific components, as well their potential uses as candidate biomarkers is yet to be determined.

Urine contains a concentrate of excreted metabolites and has traditionally been used to detect metabolites or cellular material associated with renal and metabolic disorders. For example, glycosuria indicates an abnormal use of carbohydrates and possible diabetes. The development of a metabolomic methodology (see below) makes urine samples and blood fractions the two most relevant biological fluids for determining nutritional biomarkers; in fact, urine is probably the most used biological source in nutritional studies and long-term monitoring [42]. Generally, it could be stated that the metabolome of urine reflects the *food metabolome*, that is, the content of ingested food, whereas blood samples reflect changes of the endogenous metabolome, that is, the effect of such foods on the body that ingests them. Meat intake is accompanied by high concentrations of creatine and carnitine. Creatinine is formed by creatine biodegradation in tissue and is transported via blood to the kidneys and then excreted in urine [25]. These metabolites have been proposed as biomarkers of meat intake, although they still require further validation. Urine is also a fluid that may reflect the metabolism of the microbiota [25]. Thus, the intake of foods rich in polyphenols leads to the formation of hydroxyhippuric acid and other derivatives of intestinal bacteria, which are excreted in urine [94]; similarly, urolithin A conjugates are also identified in urine, resulting from bacterial metabolism after ingesting nuts [95]. A major hurdle in the use of urine for biomarker analysis is the duration of the sampling period. Twenty-four hour urine collection is considered the gold standard but is logistically challenging especially in larger studies. Repeated sampling in combination with modelling-based approaches may provide a solution that will allow for shorter sampling periods [96]. Furthermore, relatively little is known about the stability of the urine metabolome after sampling. Immediate freezing of urine samples and prevention of freeze-thaw cycles seems to be a prerequisite for reproducible biomarker analysis [97] but is difficult in practice, especially for 24h urine collection.

Faeces are a relevant biological source for assessing the non-absorption of nutrients [98,99], the balance of a non-metabolizable ingredient (for example, nitrogen or trace metals that are excreted in bile) [100], as well as to analyse the gastrointestinal microbiota or its products [101,102,103]. Stool samples can also be used to assess biomarkers for enteropathy, which is attractive for infants for whom invasive samples are difficult to obtain [104]. A growing area of study is the detection of “volatile organic compounds” (VOCs) as a result from microbial fermentation. VOCs can be used as biomarkers and specific VOCs were shown to be associated with the intake and type of fibre [105]. Indirect calorimetry cages with hydrogen sensors are able to monitor microbial activity continuously and in real time [103]. Differences in hydrogen production by highly and lowly digestible dietary carbohydrates correspond to the abundancy of hydrogen producing bacteria [103] and to specific VOCs, including acetic-, propionic-, butyric- and valeric acids, associated with differences in microbial activity [103].

VOCs can also be sampled from breath, which is a promising tool for diagnosis of respiratory and other diseases [106,107,108]. Recently, it was also shown that analysis of VOCs in breath were different after the intake of two different infant formulae [109]. Although the observed within and between subject variation was high, breathomics data support that, with an appropriate study design and data pre-processing, specific VOC profiles have been identified and associated with fat intake from dairy drinks in comparison with a drink with the same constituents but a lower amount of fat (Hageman et al. [110]). These studies are an initial step to the use of breathomics analysis to evaluate the metabolic effects of nutritional interventions. The future use of compound-specific sensors and the non-invasive nature of VOC analysis may introduce interesting analysis in newborns and infants.

Saliva is a biological fluid that is easily collected. It is used to assess adrenal functional stress and hormone levels [111]. Furthermore, due to its minimally invasive nature, it is also one of the preferred sources for genetic analysis, as the purified DNA extracted from saliva cells allows the detection of specific polymorphic variants. Another accessible body fluid, less invasive and complex than serum or plasma are tears. Tear fluid proteomic and lipidomic analyses as well as dedicated cytokine assays are being developed to characterise ophthalmological related diseases such as dry eye disease or ocular allergies [112,113,114] and identification of putative biomarkers of systemic diseases in tear fluid is being explored [115,116,117,118].

Other easily obtainable tissues, such as nails and hair have been shown to be useful to determine long-term excess alcohol use [119], exposure to toxic metals [120] and disease related mineral status [121], although these specimens are usually bad indicators of body nutrient concentrations in healthy individuals. Hair analysis can be useful in assessing concentrations of zinc, copper, chromium and manganese for which there are no good measures of functional status [122], as well as the concentrations of cadmium and lead that can have negative biological effects [123].

Finally, obtaining solid tissue samples of potential interest in nutritional studies, such as the liver, to be used for, for example, gene expression analysis, proteomics or metabolomics, usually requires invasive biopsies, which are not easily justifiable in nutritional studies in humans. Nevertheless, adipose tissue, skeletal muscle, intestine and skin biopsies have been examined in small scale nutritional intervention studies [124,125]. As discussed above, the use of PBMCs and EVs is particularly interesting and may be a good alternative, especially in larger studies.

## 5. Types of Analysis

Two basic types of laboratory analysis are considered: static and functional. Static tests measure the current concentration of the nutrient, bioactive or biomarker in a biological sample. Examples of this type of analysis are the determination of serum iron, blood glucose, cholesterol and so forth. Circulating levels, do not necessarily reflect the amount of the substance present in body reserves or its bioavailability. Depending on the biomarker, recent intake can influence its amount in plasma, serum or any other fluid or tissue sample, although this limitation can be overcome, at least in part, by collecting the sample under fasting conditions. In contrast to static analysis, functional analysis measures a response. Although known and used for years, the pursuit of biomarkers of health has given a boost to functional measurement of a biological function that allows for a dynamic assessment to which a biomarker relates. Functional analysis allows for the quantification of the phenotypic flexibility and reflects the degree of homeostatic robustness that the individual presents [55].

Functional analyses include tests, such as the oral glucose tolerance test, for assessing the prediabetic state and insulin sensitivity and the determination of triglycerides following an oral lipid load to assess dynamic lipidaemia as a biomarker of cardiovascular risk and early detection of metabolic syndrome [126]. Recently, similar to fasting-refeeding challenges that are used in mice [127,128], a standardized liquid mixed-meal with carbohydrates, fat and protein has been proposed to assess the response of a wider set of metabolic variables in humans [129]. New type of challenges are, for example, the response to moderate level of environmental hypoxia, oxygen restriction, to assess age and body-weight induced metabolic alterations [130,131] and assessment of certain biomarkers in response to fasting, which is a potential functional analysis to characterise metabolic alterations in the obese state [132].

## 6. Nutrigenomic Approach in the Identification of Biomarkers

Global analysis techniques, known as “omics” have opened new research avenues in nutrition. Advances in DNA sequencing techniques [133] and microarray technologies [134], mass spectrometry [135] and nuclear magnetic resonance [136], among others, have facilitated simultaneous analysis of multiple parameters and have provided unprecedented insights in responses of the transcriptome, proteome and metabolome. The technological developments continue, especially with regards to DNA and RNA sequencing [137], mass spectrometry [138], single-cell omics [139] and, of course, bioinformatics [140]. Systems approaches allow obtaining a comprehensive and in-depth view of the physiology/pathology of an individual and open the possibility to explore the complex relationships between nutrition and health, particularly to investigate the role of dietary components in health maintenance or in disease development [141]. For this reason, omics platforms appear to be most suitable for the discovery and characterisation of new nutritional markers to define the nutritional status of individuals and to identify nutritional bioactive compounds responsible for beneficial health effects [142]. The identification of new biomarkers or patterns of biomarkers that link nutrition with health represents one of the major challenges of omics sciences in the nutrition field.

### 6.1. Genetic Biomarkers

Genetic biomarkers are primarily based on the determination of genetic polymorphisms, particularly of a single nucleotide (*single nucleotide polymorphisms*, SNPs). They can be determined in DNA from any biological sample containing nucleated cells, which represents an important advantage. Such biomarkers are static; thus, their determination does not change with time. Another feature of these biomarkers is that samples used can be stored and transported easily, particularly once the DNA is isolated and their determination is quick and relatively economic [53].

Polymorphic variants, which have a well-characterised biological function can be used to study the effect of a particular environmental exposure on disease risk. There are several studies using genetic variants as variables in environmental exposures. A well-known example is the lactase polymorphism 13910C>T (rs4988235), which is located on the MCM6 gene but influences the lactase gene (LCT). It is strongly associated with the persistency of lactase synthesis and hence with the tolerance or intolerance to lactose [143]. Individuals with the CC genotype usually show a physiological decline of lactase activity in intestinal cells and have difficulty in metabolising lactose. Such individuals often exhibit symptoms of abdominal pain and diarrhoea after consuming dairy products and therefore tend to consume fewer dairy products containing lactose. It has been proposed that this variant (CC genotype) in the lactase gene may act as a proxy for low milk consumption [53,144].

The study of genetic markers has advanced considerably in recent years, thanks to, among other things, the development of high-density arrays that allow simultaneous determination of thousands of genetic polymorphisms. These developments have facilitated genomic wide association studies (GWAS), which have allowed the discovery of new genes and polymorphic variants associated with intake of specific foods, such as coffee [145] or different macronutrients [146]. Likewise, genetic variants that affect the concentration of intake biomarkers have also been described, such as phylloquinone (also known as vitamin K1), which is the main circulating form of vitamin K and it reflects vitamin intake from plant [147]. Circulating phylloquinone is a biomarker of interest that has been associated with a “healthy” lifestyle and low concentrations are associated with an increased risk of various chronic diseases [148]. The description of gene variants that affect the concentration of phylloquinone may explain the large inter-individual variability in the response to the intake of phylloquinone from diet or supplements [147].

Furthermore, genetic biomarkers are crucial for determining the relationship between intermediate biomarkers (e.g., plasma lipids, fasting glucose, oxidative markers, markers of inflammation, etc.) and disease incidence (cardiovascular disease, type 2 diabetes, cancer, neurodegenerative diseases, etc.). Currently there are hundreds of SNPs consistently associated with different phenotypes of nutrition-related diseases [149,150]. Therefore, in nutritional epidemiology studies, determination of the most relevant genetic polymorphisms associated with phenotypes of interest is important in order to establish a reliable association between diet and disease. This is particularly relevant when inter-individual variation has been associated with the presence of certain gene variants, which may influence the correct assessment of nutritional status.

In addition to these considerations, genome-associated individual variability can be relevant in the proper assessment of micronutrient status, which can have a narrow safety range between safety and toxic doses [54,151] or modulate its bioavailability [152].

A representative example is selenium status, which is associated with an increased risk for various chronic diseases when it is low [153]. Biological effects of selenium are largely mediated by a family of around 25 proteins, which contain at least one selenium containing amino acid, selenocysteine [154]. Evidence suggests that individual requirements for selenium differ because of polymorphisms in selenoprotein encoding genes. Synthesis of selenoproteins is actively regulated by the selenium status and its expression is reduced in a hierarchical process to facilitate the expression of others when selenium availability is limited [155]. Glutathione peroxidases (GPX), which are involved in antioxidant function, and Selenoprotein P (SEPP), which is responsible for the selenium transport and supply to tissues, together constitute half of the selenium in blood. Optimal selenium intake is associated with optimal expression of all selenoproteins and when selenium is consumed above needs, the excess is excreted, since there is no regulated reserve pool of selenium [156]. As a consequence, genetic variation needs to be considered in assessment of selenium status. For example, individuals with GPX1 679T/T alleles show lower plasma selenium levels than those with C/C alleles, because this variant also accounts for differences in urinary excretion of selenium. Individuals with the SEPP1 24731 A/A genotype show higher plasma SEPP1 levels in comparison with those with the G allele. Gender and BMI also contribute to variation in biomarkers of selenium function [156]. Zinc constitutes another example of an essential micronutrient, with fundamental roles in human biology, of which the nutritional status is associated with genetic background. For example, a number of zinc transporters coordinate zinc homeostasis. Insulin metabolism in pancreatic β-cells requires zinc and a polymorphism in the zinc transporter SLC30A8 has been associated with increasing risk of developing type 2 diabetes [151]. In fact, total zinc intake shows an inverse relationship with fasting plasma glucose in individuals carrying the glucose-raising A allele. Various lines of evidence support the concept that zinc recommendations may benefit from being personalized [151].

### 6.2. Epigenetic Markers

The term epigenetics is used to describe a variety of changes in the genome that do not involve changes in the DNA sequence but concern other chemical modifications that can result in differential gene expression. Unlike genetic variations, which are largely fixed, epigenetic modifications are temporal, ranging from stable within a generation to being the result of immediate adaptation to the environment or metabolism. The main epigenetic mechanisms include DNA methylation, histone modifications, mainly site-specific methylations and acylations [157].

DNA methylation is an epigenetic modification, which occurs in a cytosine-phosphate-guanine dinucleotide (CpG) and involves the addition of a methyl group at the 5 position of cytosine residues in CpG islands. This modification provides marks in the genome that establish whether the genes are activated transcriptionally or silenced. Hypomethylation or hypermethylation of specific islands has been associated with several disease phenotypes such as cancer, obesity or type 2 diabetes, among others [158] or with the protection against some diseases [159]. Studies show that diet can affect the methylation of certain DNA sites and that these changes in methylation are dynamic. For example, CpG methylation sites have been associated with the intake of EPA and DHA from marine sources [157]. Furthermore, a diet rich in conjugated linoleic acid and calcium, which promotes weight loss in rodents, has been associated with changes in the degree of methylation of lipid metabolism-related genes, such as fatty acid synthase and stearoyl-CoA desaturase [160].

Epigenetic modifications caused by changes in DNA methylation status represent one of the mechanisms that may explain the effects of metabolic programming of the offspring during the perinatal period. For example, intrauterine growth retardation in rats was shown to block expression of *Pdx1*, a pancreatic gene that mediates the glucose responsive transcription of the insulin gene, in the offspring [161]. While this modification seems permanent in the first generation offspring, modifications at other epigenetic marks were shown to be reversible. For example, skeletal muscle DNA methylation of the orphan nuclear receptor *Nr4a1*, which is linked to insulin sensitivity, was shown to be programmed by the mouse maternal diet and was subsequently modulated in offspring by voluntary exercise [162]. Animal studies have also shown that changes in maternal intake during pregnancy that affect the availability of methyl donors can alter the epigenetic pattern of certain regions of the genome (metastable epi-alleles) in the early embryo that are stable in different tissues, causing permanent phenotypic variation in offspring [163]. In humans, although studies are still scarce, there is increasing evidence showing that perinatal nutrition may trigger persistent changes in DNA methylation [164]. For example, it has been shown that variations in methyl donor intake (associated with seasonal differences in diet: the rainy (‘hungry’) season and the dry (‘harvest’) season) in women of a rural population of Gambia during conception predict the methylation patterns of metastable epialleles in offspring [165,166]. Notably, 13 biomarkers, have been identified consisting in key micronutrients involved in one-carbon metabolism, whose levels in maternal plasma may predict DNA methylation changes at metastable epialleles in DNA from hair follicles and lymphocytes in infants postnatally [166]. Changes in DNA methylation have also been reported in individuals with prenatal exposure to famine, such is the case of the Dutch Hunger Winter at the end of World War II [167,168]. Concretely, six decades later, DNA methylation levels for several loci—including insulin like growth factor 2 (*IGF2*), interleukin 10 (*IL10*), leptin (*LEP*) and so forth—were found to be altered in these individuals compared with their unexposed same-sex siblings and changes were generally found when exposure to famine occurred during the periconceptional period, suggesting that the methylome is more susceptible to alterations at early stages of development [167,168].

Epigenetic regulation likely involves complex interactions between various nutrients. For example, vitamin C is an essential co-factor for multiple demethylases that regulate DNA and histones methylation [169]. These demethylases are also sensitive to reactive oxygen species (ROS) and tricarboxylic acid cycle (TCA) metabolites. They depend on alpha ketoglutarate and are inhibited by succinate and fumarate. Both the electron transport chain activity, as a source of ROS and TCA cycle are dependent on the status of various B-vitamins [170]. Vitamin C, B-vitamins and substrate fluxes thus interact in epigenetic regulation. Despite rapid progress, nutritional epigenetics is still in its infancy and many more studies are needed in order to establish epigenetic markers as new biomarkers of intake or of nutrition related health/disease.

While attention has initially been focused on DNA methylation, histone modifications are currently emerging as nutrition-relevant epigenetic modifications. For example, methylation of histone 3 at lysine 4 of the histone tail has been associated with undernourishment in young children [171]. In particular histone acylation, modifications by acetyl and other acyl groups, directly link epigenetically regulated gene expression to metabolic activity, flux and status [170]. Histone acylation levels are determined by the balance between available acylation substrates, acylase levels and activity and de-acylase levels and activities. Acylation substrates can be provided by TCA cycle, by diet and by microbial fermentation [172]. To illustrate further nutritional complexity, beta-oxidation as well depends on the status of various B-vitamins [170]. Furthermore, histone deacetylase class III members, the sirtuin family of NAD^+^-dependent deacylases, are dependent on vitamin B3 [173]. SIRT1 levels and histone de-acetylase activity were also shown to be affected by supplementation with resveratrol [174,175].

### 6.3. Transcriptome Markers

Transcriptomics allows us to study the transcriptome, either individually for each specific gene of interest (generally using real-time RT-PCR techniques) or for the analysis of multiple genes or the complete set of genes expressed simultaneously in a tissue. The use of DNA-microarrays has been established as highly robust technology for transcriptome analysis [134] but RNA sequencing (RNA seq) is now rapidly emerging as an alternative [176]. The advantage of RNA seq over microarrays is that a larger spectrum of RNAs is covered, which potentially can provide more functional information [177], although in practice, most of the attention is focused on annotated transcripts, which are also well represented on state-of-the art DNA microarrays. The use of RNA seq requires more complex bioinformatics [178,179] and technical robustness can still be improved, especially for small samples [180]. However, with the estimated further improvement of sequencing technologies and associated decrease in costs, RNA seq is expected to become the future standard. By analysing the transcriptome, we can investigate how exposure to different diets, specific foods or components of diet, affects the expression of specific genes or more globally, the complete transcriptome. Global transcriptome analysis has been a major tool in unravelling the molecular mechanisms of disease and has facilitated the search and identification of biomarkers of health. The analysis of gene expression using appropriate bioinformatics tools allows a more profound understanding of metabolic pathways and regulatory networks and is helping to identify biomarkers for diagnosis and prognosis, as well as potential targets for medical and nutritional intervention. Moreover, transcriptomic studies have improved the understanding of the complex interplay between genetic and environmental factors, such as lifestyle and nutrition factors [181,182].

The transcriptome is not the same for all cells in the body but varies depending on the tissue and time of life, which can complicate the collection and use of such biomarkers. Moreover, as discussed above, obtaining samples from tissues of interest, such as the liver, muscle or adipose tissue, may be a limiting point in human studies, because it involves performing invasive biopsies. In this regard, blood cells (PBCs), either total cells or the mononuclear cell fraction of peripheral blood (PBMCs), provide an attractive alternative because they can be obtained relatively easily and in sufficient quantities by minimally invasive techniques [132,183]. These cells travel throughout the body and are able to sense and respond to internal and external signals. They have been proposed as a source of transcriptomic biomarkers of health and disease, since their gene expression profile reflects in part the expression profile which occurs in other tissues, particularly liver, muscle and adipose tissue, which evolutionary derive from the same body compartment as PBMC [68,69,70,71]. Hence, changes occurring in gene expression in such cells may be indicative of the physiological and pathological state of the body and have a predictive component [184]. For this reason, the transcriptional profile of PBCs represents a very useful tool for evaluating the physiological and nutritional effects of food or its components [66].

Several studies show the association between diet and transcriptional profiling of PBCs. For example, different gene expression profiles in PBCs in healthy individuals have been described according to dietary patterns: a “Prudent” dietary pattern - with high intakes of fruits and vegetables and whole grain products and low intakes of refined grain product - compared with a Western dietary pattern [185]. Also, changes in the PBC transcriptome have been observed after consumption of diets rich in omega-3 polyunsaturated fatty acids (omega-3 PUFAs) or other dietary modifications [66,186]. Moreover, differences in the expression of specific genes in PBCs have been described in children related to the frequency of sugary food (*TAS1R3*) or high-fat (*UCN2*) consumption [187]. Hence, expression levels of these genes were suggested as potential biomarkers of the frequency of intake of specific foods, which could complement data from questionnaires [187]. Notably, in this study, it was shown that transcript levels of *TAS1R3* in PBCs were related with changes in BMI and fat-mass after a two-year follow-up period in children, with low expression levels of this gene being related with increased fat accumulation overtime, being a more accurately measurement than the reported consumption of sugary foods [187]. Similarly, changes in expression levels of specific genes in PBCs have also been described in children depending on the metabolic status and therefore they have been proposed as potential biomarkers of risk for insulin resistance or dyslipidaemia associated with obesity [73]. Such biomarkers, although promising, still need to be validated in other studies.

Considering the effects of certain gene variants and epigenetic modifications on the level of gene expression, it is relevant to integrate transcriptome studies with genomics and particularly epigenomics, since the epigenomic machinery is highly sensitive to metabolic cues [188].

#### 6.3.1. Non-coding RNAs

Non coding RNAs (ncRNAs), both microRNAs (miRNAs) and long-chain non-coding RNAs (lcRNAs) have emerged as regulators of mRNA transcription. For example, non-coding RNAs have been shown to regulate a wide array of diet-induced obesity associated processes, including adipogenesis, adipokine secretion, inflammation, glucose metabolism, lipolysis, lipogenesis, white adipose tissue (WAT) hypoxia and WAT browning [189]. While the regulatory role of lcRNAs (>200 bp) is still emerging [190] and is poorly investigated in the field nutrition, miRNAs have emerged as crucial epigenetic regulators of many processes related to nutrition, including nutritional regulation of disease related pathways [191]. MiRNAs are small RNA of 18-25 nucleotides in length, which regulate expression of their respective target mRNAs post-transcriptionally. Dietary modulation of miRNA expression has been shown influence various diseases, such as type 2 diabetes, obesity or hepatic steatosis [192]. Food components have been shown to modulate the expression of miRNAs [193]. For example, diets rich in conjugated linoleic acid or PUFAs have been shown to modulate specific miRNAs [194,195]. Some miRNAs were associated with the dietary exposure (PUFAs), while other were associated with markers of inflammation and metabolic health [195].

MiRNAs have also been shown to enter the body from dietary sources, including plant foods and cow milk [193] and a database has been established for the presence of miRNAs in various dietary sources [193]. A number of studies have documented potential cross-kingdom communication by diet/plant-derived miRNAs, although some contradictory data have also been collected and, up to now, current controversy exists concerning the exogenous transfer and bioavailability of exogenous miRNAs [118]. This is especially true for plant-derived miRNAs, while stronger scientific evidence is available for cow milk miRNAs [196,197], in particular for transfer over the relatively immature intestine of newborns. Furthermore, growing evidence indicates that miRNAs expressed in breast milk may reflect maternal diet and nutritional status and, therefore, may influence offspring phenotype [198]. Thus, specific miRNAs have potential as biomarkers of effect, exposure and intake.

### 6.4. Proteomic Markers

The proteome is the set of proteins that is or can be expressed by a genome, in a cell, tissue or organism at a certain time. As is the case with the transcriptome, the proteome is dynamic and varies with the cell type and its functional status. Bioactive food components usually have a limited influence on the genome, while the effects on the transcriptome and proteome are generally greater.

Generally, readily accessible body fluids (blood, saliva or tears) contain proteins of physiological and diagnostic importance. They are widely used in clinical tests for diagnosis and prognosis of diseases and to follow their evolutions [199]. Tears, for example, are a complex biological fluid and the tear proteome has been suggested as a relevant source for clinical diagnostic markers [112]. Most human diseases involve changes in the expression of normal proteins or the creation of abnormal proteins, that perturb physiology. In many cases, these proteins may appear in blood or other biological fluids, thereby providing an easy access biomarker which can offer information on the disease process. Proteomics also allows the identification of changes that occur in response to diet. For example, in an animal model, the application of proteomic studies combined with physiological studies has provided new insights into the mechanisms by which dietary interventions with different sources of fatty acids (fish oil, conjugated linoleic acid and elaidic acid) regulate lipid metabolism and other related pathways and determine changes in lipemia and insulin concentration [200].

The use of proteomic techniques for the identification of new biomarkers has generally been limited by the characteristics of proteins and the availability of suitable techniques. The methodology of two-dimensional electrophoresis, initially used in proteomics, has inherent disadvantages: (i) bias towards the most abundant changes, giving a poor resolution for low abundance proteins; (ii) inability to detect proteins with extreme properties (very small, very large, very hydrophobic or acidic or basic); (iii) difficulty in identifying proteins, since it is time consuming and costly [201]. Recent advances in mass spectrometry, with greater sensitivity, specificity and resolution capabilities, make it feasible to use this technology in order to detect, identify and quantify proteins in blood and other biological fluids. The sampling of larger number of individuals has shown that biomarker discovery with the use of mass-spectrometry and isobaric tagging provides robust and consistent biological results [202]. Furthermore, studies also show effects of gender and phenotype, in particular age and fat mass, which has to be taken into account for diagnostic applications [203]. A promising proteomic approach is the protein microarray technology, which can be used to detect changes in expression and post-translational modifications of hundreds or even thousands of proteins at the same time. Its advantages include high sensitivity, good reproducibility, quantitative accuracy and possibility of parallel individual determinations. These microarrays have opened new possibilities for the study of the molecular mechanisms underlying the interactions between nutrients and genes [201]. However, it should be noted that, compared to DNA microarrays, protein microarrays are still at an early stage of development but its multiple applications are gradually being developed, expanded and improved. In addition to post-translational enzymatic modification, proteins may also be modified non-enzymatically as a consequence of metabolic fluxes. For example, advanced glycation end products (AGEs) are formed upon reactions of sugars or sugar metabolites with proteins. Using mass spectrometry, specific AGEs have been identified as potential biomarkers for changes in glucose metabolism related to diabetes and/or age [204].

The use of proteomics in nutritional research has not lived up to its expectations but gradual progress is being made. It has, for example, been examined as a tool to evaluate the effects of dietary regimens in cancer treatment [205]. An interesting example is the use of proteomics to support the beneficial effects of purple vegetables, carrots and potatoes on metabolic health [206]. The collection of information on proteins and peptides, their cellular locations and functions, along with their expression patterns in different tissues and cells, provides powerful material for defining hypotheses regarding potential biomarkers in serum/plasma, prior to validation with specific tests [207]. The creation of databases of proteins present in blood is expected to help identify new biomarkers [208].

### 6.5. Metabolomic and Lipidomic Markers

Metabolomics or metabolite profiling, can be defined as an analysis or screening of small metabolites present in samples of biological origin [209]. Metabolomics has undergone major progress in the last two decades, mainly through significant innovations in instrument technology, especially mass spectrometry and gas and liquid chromatography techniques, together with bioinformatic tools and software [209,210]. In metabolomics, targeted and untargeted approaches can be carried out. Targeted metabolomics allows the analysis of a defined set of known metabolites with similar structures (e.g., amino acids, fatty acids, acylcarnitines, phytochemicals, etc.) and is generally a quantitative tool. This approach is commonly aimed at answering specific biochemical questions or hypothesis that motivate the investigation of one or more related pathways [210]. For example, a targeted approach has allowed the identification of a set of five amino acids (isoleucine, leucine, valine, tyrosine and phenylalanine) whose fasting levels strongly predicted future diabetes [211]; or a set of metabolites (Leucine/Isoleucine and glycerol) whose response after an oral glucose tolerance test might be predictive of insulin sensitivity [212].

Untargeted metabolomics (also referred to as “shotgun” metabolomics) consists in the unbiased screening of metabolites in biological specimens and is generally used for global metabolite profiling with the intention of comparing patterns of metabolites among different groups [30]. This approach is capable to detect thousands of independent spectral features in a biological sample [209]. However, unlike target metabolomics, only a part of the detected peaks (about one-third of them, as an estimation) are included in databases and metabolite repositories and can be unequivocally linked to a specific chemical structure. Untargeted metabolomic studies are generally not driven by hypothesis but are rather hypothesis generating [210].

Metabolomics strategies, both targeted and untargeted, have clearly contributed to biomarker discovery of the last years and many reports provide the proof-of-principle of metabolomics being a key tool for nutrition research [30,213,214]. The comprehensive metabolite profiles (metabolome) can provide an overview of the metabolism with a level of description that transcends genetic information and more closely reflects the ultimate phenotype, thus helping to connect genotype to phenotype at the molecular level [210]. If mechanistically substantiated, changes in the metabolome may be used to improve disease risk estimates in epidemiological studies. Indeed, metabolomics is already being successfully used in the identification of food components and their metabolites in biological fluids [213,215]. Thus, metabolomics has allowed to define dietary exposures, for example the intake of meat, fish, dietary pulses and so forth. [213,216,217,218], health status [219,220] and to examine the result of nutritional intervention strategies [221,222,223].

Metabolites present in blood or other biological samples, not only reflect dietary exposure but also metabolic processes, including the modifying effects of genetic variation and intestinal microbiota [30]. Concerning intestinal microbiota, it is of key importance to understand how changes in the microbiota composition affect its functionality for interpretation of possible health outcomes [224]. Integrate of serum metabolome and microbiota composition data is instrumental in linking functionality to health [225,226]. The Mediterranean diet was, for instance, shown to modify gut microbiota with functional consequences both in the microbiome and the host metabolome, associated with reduction in disease risk [227]. Diet and food components can shape the composition of the gut microbiota. For example, highly and lowly digestible fibres differentially affect gut microbiota as measured by hydrogen production and parallel changes in hydrogen producing bacteria [228]. Not only macronutrients, depending on their percent composition in the diet, are important for modulating the composition of gut microbiota and their functionality [227] but also specific bioactive compounds, such as flavonoids, whose bioconversion is highly variable dependent on the microbiome composition, thus influencing their biological activity and the possible physiological and health outcomes [229]. Therefore, the diet modulates the microbiota but the microbiota composition also modulates the effects of diet and food components and hence the response to diet. In this sense, the analysis of the microbiome of the individuals may help to better interpret the response to dietary patterns/components and has been proposed as promising for the search of biomarkers to predict individual responsiveness to diet [227]. Thus, the use of microbiota composition as well as the integrate analysis of the metabolome and the microbiome as biomarker of dietary assessment have biomarker discovery potential [225,226].

Lipidomics is defined as the metabolomic analysis of lipids. It can be considered as a subfield in metabolomics, since the different solubility properties of lipids compared to other metabolites often determines their separate analysis [230]. Lipidomics has become the primary tool for the identification and diagnosis of inborn errors of lipid metabolism [231]. It is now increasingly used in nutritional studies, especial since commercial companies can deliver lipidome profiles in a robust manner, with increasingly competitive prices. Lipidomics is being used for effect analysis [128,232] but also to monitor dietary exposure [233,234] and the relationship between food intake and health parameters [234,235]. Lipidomics has also been useful to provide some insights into metabolic pathways by which food exposure may exert its health effects [235].

In recent years, metabolomics is being introduced in large cohort nutritional studies, with promising results. The improvement of technologies, which are progressively more powerful and sensitive and the growing availability of comprehensive databases (including food components and their metabolic derivatives) are helping this process. For example, a metabolomic study has identified 39 known metabolites in serum which correlate with a total of 13 dietary groups, including citrus fruits, green vegetables, red meat, shellfish, fish, peanuts, rice, butter, coffee, beer, spirits, total ethanol and multivitamins [236]. As an example, strong associations between consumption of citrus and stachydrine, coffee intake and trigonelline (N-methyl-nicotinate) and quinine or alcohol consumption and ethyl glucuronide, have been described [236].

In addition to the studies carried out in serum samples, metabolomic studies have also been done using urine samples, which have revealed the existence of markers associated with intake, for example, with the consumption of meat (1-methylhistidine, O-acetylcarnitine) [35,41], vegetables (phenylacetylglutamine) [41], citrus (proline betaine), oily fish (1-methylhistidine), coffee (dyhydrocaffeic acid derivatives) [27] and tomato juice (hydroxylated and sulfonated metabolites of esculeogenin B) [34].

In general, metabolome based biomarkers, along with others identified using the previously described omics techniques, are of great interest in nutrition, because they can be used to monitor intake in epidemiological or intervention studies, complementing the results of dietary questionnaires. Moreover, the development of fast and affordable tests for relevant biomarkers of food intake could also be of interest to routinely assess nutritional deficiencies and imbalances in the population.

## 7. Empowering Citizens to Monitor and Follow a Healthy Diet

The future of nutrition is moving towards the possibility of carrying out real personalised nutrition, the emerging concept of “precision nutrition,” which may be achieved as a result of a rigorous nutrigenomic analysis that considers the genetic makeup of the individual, its epigenetic modulation and its molecular phenotype [59].

The health of an individual depends on the information contained in its genome and how it is interpreted throughout its life (epigenome, methylome, transcriptome, proteome and metabolome). The dynamic evaluation of physiology and the health status via an integrated analysis of all these factors is what is called an integrated personal omic profile (iPOP) [237]. Although we are still far from being able to define and use iPOPs, the first description that exists of the iPOP for a single individual has shown the enormous potential of omics integration in medical research, in monitoring health status and personalised medicine [237]. The iPOP is a preventive and diagnostic tool because it can follow and to a certain extend predict, the evolution of health status and evaluate metabolic robustness. Furthermore, it might also help to improve the assessment of disease risk and provide high diagnostic accuracy, monitoring of disease, targeted therapies and understanding of the associated biological processes. Clearly, the availability of such information requires powerful tools for integration and interpretation. Hence, it is necessary to develop algorithms that enable a holistic understanding of all the events that shape and participate in defining the health status of individuals throughout their life, information that could be collected by conducting longitudinal iPOPS associated with crucial stages of life.

This concept, which would initially define the health status and the metabolic and endogenous responses of an individual, would allow to identify certain exogenous factors, including dietary factors that have the potential to modify the iPOP in an integrated manner, allowing to establish functional nutritional behaviours towards improved health for the individual. The enormous development of information technology, in terms of algorithms and appliances, can be instrumental in iPOP implementation. The acquisition of food intake information by the consumer via mobile appliances can be translated by image recognition software allowing for efficient identification of food ingredients. Combining this information with a personal integrative nutritional biomarker profile, would optimally help providing more adequate, precision nutrition recommendations. Additional physiological information, for example to monitor glucose levels, may be provided by wearables. The use of specifically designed platforms, for example, user-friendly mobile applications, capable of integrating all this available information and translating it into specific outcomes, is expected to help empowering citizens to have healthier optimal behaviours and life-style adaptations
